# Inhibition of COX-2 signaling favors *E. coli* during urinary tract infection

**DOI:** 10.1186/s12950-023-00356-9

**Published:** 2023-09-11

**Authors:** Soumitra Mohanty, Ciska Lindelauf, John Kerr White, Andrea Scheffschick, Ewa Ehrenborg, Isak Demirel, Hanna Brauner, Annelie Brauner

**Affiliations:** 1 Department of Microbiology, Tumor and Cell Biology, Karolinska Institutet, Stockholm, Sweden; 2 Division of Clinical Microbiology, Karolinska University Hospital, Stockholm, Sweden; 3 Department of Medicine, Solna, Stockholm, Sweden; 4 Center for Molecular Medicine, Karolinska Institutet, Solna, Sweden; 5 Cardiovascular Medicine Unit, Department of Medicine, Center for Molecular Medicine at BioClinicum, Karolinska University Hospital, Karolinska Institutet, Stockholm, Sweden; 6 iRiSC - Inflammatory Response and Infection Susceptibility Centre, Faculty of Medicine and Health, School of Medical Sciences, Örebro University, Örebro, Sweden; 7 Dermato-Venereology Clinic, Karolinska University Hospital, Stockholm, Sweden

**Keywords:** *E. coli*, COX-2, Antimicrobial peptide, Cytokines, Urinary tract infection

## Abstract

**Background:**

To avoid the overuse of antibiotics, non-steroidal anti-inflammatory drugs (NSAIDs), acting via cyclooxygenase (COX) inhibition, have been used to reduce pain and as an alternative treatment for uncomplicated urinary tract infections (UTIs). However, clinical studies evaluating NSAIDs versus antibiotics have reported an increased risk of acute pyelonephritis. Therefore, we hypothesized that COX inhibition could compromise the innate immune response and contribute to complications in patients with uncomplicated UTI.

**Results:**

We here demonstrate that in particular COX-2 inhibition led to decreased expression of the antimicrobial peptides psoriasin and human β-defensin-2 in human uroepithelial cells. Psoriasin expression was altered in neutrophils and macrophages. COX-2 inhibition also had impact on the inflammasome mediated IL-1β expression in response to uroepithelial *E. coli* infection. Further, COX-2 inhibition downregulated free radicals and the epithelial barrier protein claudin 1, favoring infectivity. In addition, conditioned media from COX-2 inhibited uroepithelial cells infected with *E. coli* failed to activate macrophages.

**Conclusions:**

Taken together, our data suggests an adverse innate immune effect of COX-2 inhibition on uroepithelial cells during UTI.

**Supplementary Information:**

The online version contains supplementary material available at 10.1186/s12950-023-00356-9.

## Introduction

Urinary tract infections (UTIs), often caused by *E. coli* [1], are one of the most common bacterial infections, second only to respiratory infections with respect to antibiotic prescription [2]. During UTI, pain associated with voiding is a major symptom and considered to be influenced by an inflammatory reaction. As a result, non-steroidal anti-inflammatory drugs (NSAIDs) are used to dampen the release of pro-inflammatory cytokines and chemokines as well as the pain triggered by elevated prostaglandin E2 levels [3, 4]. Since 50–60% of women experience at least one UTI in their lifetime and 25–30% of these have recurrent infections, the disease has great impact on the patient’s quality of life [5]. Although antibiotics are the drug of choice for treating UTI, the increasing antibiotic resistance amongst isolates of *E. coli* causing UTI [6] demands alternative treatment strategies.

For many patients, UTI is a self-limiting disease [7]. Therefore, in an attempt to avoid unneccesary antibiotic use, NSAIDs and similar drugs have been tried as an alternate regimen for mild cystitis. An earlier clinical trial supported this, by suggesting non-inferiority of ibuprofen compared to antibiotics for the treatment of symptomatic uncomplicated UTI [8]. Such change of treatment would decrease the antibiotic consumption and reduce the selective pressure for bacteria to develop resistance. In contrast, recent clinical studies have instead shown that NSAIDs were less effective than antibiotics in treating UTI and were associated with more symptoms and with a longer disease duration. Specifically, there was an increased risk of developing acute pyelonephritis in NSAIDs treated patients [9–12].

Antimicrobial peptides (AMPs) are part of the innate immune response and amongst the body’s first line of defense towards invading pathogens. AMPs include cathelicidin [13], β defensin-1 [14], the RNase superfamily [15, 16] and psoriasin [17], residing in epithelial and immune cells. NSAIDs mainly act by inhibiting cyclooxygenase (COX)-1 and 2 signalling [18]. Although the use of NSAIDs over antibiotics remains alluring, empirical evidence regarding the possible influence of NSAIDs on the innate immune defense during *E. coli* infection has not been elucidated. We hypothesize that NSAIDs will have immunocompromised effect on the innate immune response during acute infection that in turn will affect the rate of *E. coli* infection. Therefore, in this study, we aim to investigate the impact of COX-2 inhibitor and underlying relevant mechanisms on the possibility of bacterial infection in the urinary tract.

## Materials and methods

### COX inhibitors

Specific COX-1 inhibitor, SC-560 (1µM) and COX-2 inhibitor, SC-236 (5µM) (Sigma) were dissolved in DMSO and used in all the experiments. Equivalent volume of DMSO alone served as the vehicle control. Above used concentrations were not cytotoxic for human uroepithelial cells, 5637 and T-24 as confirmed by XTT cell viability assay [19] (Supplementary Fig. 1).

### Bacterial strain

Uropathogenic *E. coli* strain CFT073 [20] was used for in vitro and in vivo experiments. Bacteria were grown over night on blood agar plates at 37^°^ C, resuspended in 1X phosphate-buffered saline (PBS), measured spectrophotometrically and confirmed by viable count.

### Mouse model of UTI

Mice experiments were approved by the Northern Stockholm Animal Ethics Committee, and experiments were carried out according to the guidelines of the Federation of Laboratory Animal Science Association and in compliance with the Committee’s requirements. Female C57BL/6j mice were obtained from Janvier Laboratories. Animals were housed following standard procedures. 10 mg/kg of SC-236 were administered once daily via oral gavage at least 7 days prior to the infection. Mice were anaesthetized using isoflurane and transurethrally infected with 0.5 × 10^8^ colony-forming units (CFU) of *E. coli* CFT073 in 50 µl of PBS. After 24 h infection, mice were sacrificed and respective urinary bladders were aseptically removed, cut open and washed with PBS to remove urine and non-adherent bacteria. To determine the total bacterial load, adhered and intracellular bacteria, bladders were homogenized in 1 ml of PBS, serially diluted and plated on blood agar plates.

### Cell lines and culture conditions

Human uroepithelial cells, 5637 (HTB-9, American Type Culture Collection) and T-24 (HTB-4, American Type Culture Collection) were cultured in RPMI 1640 and McCoy´s 5a medium (Life Technologies) supplemented with 10% fetal bovine serum (FBS; Life Technologies), respectively. Human uroepithelial cells, TERT-NHUC (kindly provided by M. A. Knowles, Leeds, UK) were grown in EpiLife medium (Life Technologies) supplemented with 1% of human keratinocyte growth supplement (HKGS, Life technologies). Primary neutrophils isolated from human blood were cultured in RPMI 1640 with 10% FBS. THP-1 differentiated macrophages (TIB-202, American Type Culture Collection) and human promyeloblasts HL-60 (CCL-240, American Type Culture Collection) were cultured in RPMI 1640 with 10% FBS and were differentiated with 150 ng/ml of PMA (24 h) or 1.3% of DMSO (for 7 days), respectively. Human NK cells, NKL (kind gift from Dr. M. J. Robertson (Indiana University School of Medicine, Indianapolis, IN) [21] were cultured in RPMI 1640 with LGlutamine supplemented with 100 U/ml of IL-2 and 10% FBS. All the cells were cultured at 37^°^C and 5% CO_2_.

### Primary neutrophil isolation

Human neutrophils were prepared from buffy coats obtained at the Karolinska University Hospital blood central. According to Swedish law, the use of anonymized buffy coats does not require specific ethical approval. Neutrophils were isolated using a hyperosmotic Percoll (GE Healthcare) solution and subsequent centrifuged (580 x g, 15 min) using standard protocol [22]. Blood separated out in 6 distinct bands. Neutrophil layers were carefully collected, and10 ml HBSS without Ca^2+^ and Mg^2+^ were mixed by inverting the tube and centrifuged at 350 x g for 10 min. 2 ml RBC lysis buffer was added to the pellet, vortexed slowly and centrifuged 250 x g for 5 min. The above described lysis step was repeated to remove the RBCs. Further, cell pellet was washed with HBSS without Ca^2+^ and Mg^2+^, centrifuged 250 x g for 5 min to obtain the neutrophils.

### Cell infection assays

Cell experiments were carried out in 24 well cell culture plates. TERT-NHUC cells were cultured in Primaria (BD). All other cell lines T-24, 5637, differentiated HL-60 (dHL-60) and differentiated THP-1 (dTHP-1) were cultured in Costar plates. Cells were grown in the presence of 5µM SC-236 for 24 h prior to the infection. After 24 h, old media was removed and replaced with fresh media supplemented with 5µM of SC-236, without antibiotics and serum. 10^6^ CFU/ml (MOI 5) or 2 × 10^6^ CFU/ml (MOI 10) of *E. coli* CFT073 were added to nearly confluent pre-treated cells and incubated in 37 °C at 5% CO_2_ and 80% humidity. At pre-set time points like 15, 30, 60 and 120 min, cells were washed once with PBS and harvested for further analysis.

### Bacterial survival assay

5637 and dTHP-1 cells were infected with MOI 5 *E. coli* in 100 µl of PBS per well, centrifuged for 1 min at 350 g. For assessment of bacterial adhesion, cells were infected for 30 min and washed with PBS. In survival assays, 5637 cells were washed with PBS after 2 h infection to remove non-adherent bacteria and supplemented with fresh medium with or without 5µM of SC-236 for another 2 h. However, for dTHP-1, 100 µg/ml of gentamicin was used for 30 min to kill the extracellular bacteria. In COX-2 inhibited cells, medium was supplemented with 5µM of SC-236 throughout the entire experiment. At indicated time points, cells were lysed with 200µL of ice-cold 0.1% Triton-X-100 in PBS (pH-7.4) and scraped. Cell lysates were serially diluted in PBS and plated on blood agar plates. Cells treated with 5µM of SC-236 were compared to DMSO treated cells. Rate of adhesion and survival were calculated by number of adhered or intracellular bacteria in relation to the total number of bacteria from the same experiment.

#### Free radical formation assay

5637 and dTHP-1 cells were infected as described earlier after incubation with 5µM of SC-236 for 24 h. Supernatants were collected and mixed with equal volumes of Griess reagent (Invitrogen) based on the manufacturer’s protocol. Optical density was measured at 550 nm, and free nitrite was evaluated and normalized to DMSO treated control cells. For total ROS analysis, 10 µM DCFH-DA (Sigma) was added to the cells and were incubated at 37° C and 5% CO_2_ for another 2 h. Fluorescence intensity was measured at excitation 485 nm and emission 530 nm (Fluostar Omega). Similarly, for mitochondrial ROS analysis after 1 h of *E. coli* infection, cells were first washed with 1× Hanks’ Balanced Salt Solution (HBSS), then 5 µM Mitosox (Life Technologies) was added and left for 30 min, then live cell imaging was done using Zeiss LSM 700 to measure the mitochondrial ROS. Fluorescence intensity was quantified using ImageJ software.

### Conditioned media-activation assay

T-24 cells were pretreated with 5µM of SC-236 for 36 h, followed by 2 h infection with *E. coli* CFT073. Conditioned media was harvested and used for stimulation of dTHP-1, dHL-60 and NKL cells with 1:2 ratio. dHL-60 and NKL cells were harvested after 6 h activation whereas dTHP-1 cells were harvested after 2 h. Early activation marker CD11b (BD Biosciences), CD63 (BD Biosciences) and psoriasin (Santacruz) were used to stain dHL-60 cells whereas M1 activation marker CD86 (BD Biosciences) was used for dTHP-1 cells using standard recommendation of the manufacturer. For NKL cells, Golgi plug/Brefeldin (final concentration 5 µg/ml, for intracellular proteins) was dispensed after 1 h of stimulation and continued for a total of 6 h activation. Thereafter, NKL cells were washed with PBS + 1% fetal calf serum, live/dead marker (Thermo Scientific) was added and incubated for 30 min at 4 °C. Cells were washed and thereafter fixed and permeabilized with 1X fixation/permeabilization solution (BD Biosciences), Cells were thereafter washed in perm/wash buffer and stained with anti-Granzyme B (BD Biosciences, diluted in perm/wash buffer, for 30 min at 4 °C in the dark. Finally, cells were washed in perm/was buffer, dissolved in PBS and data acquired at BD FACS Fortessa and analyzed in FlowJo software.

### Total RNA isolation and quantitative real-time PCR (qPCR)

Total RNA was extracted using the RNeasy Mini kit (Qiagen) according to the manufacturer’s protocol. The concentration and purity of RNA was determined with nanodrop, and up to 0.5 µg of RNA was transcribed to cDNA using the High-Capacity cDNA Reverse Transcription Kit (Applied Biosystems). Expression of target genes was analyzed using SYBR Green reagent in Rotor-Gene PCR cycler (Corbett Life Science) with gene specific primers (Supplementary Table 1). Relative expressions of target genes were presented as 2^−∆CT^ and fold change as 2^−∆∆CT^ compared to uninfected or non-treated control.

### Immunofluorescence of cells

Cell infections were performed after 36 h of 5µM of SC-236 treatment as described earlier. After infection, cells were fixed in 4% PFA for 15 min at room temperature and permeabilized with 0.1% Triton X-100 in PBS. Thereafter, cells were blocked for an additional 60 min with the 5% BSA. Cells were stained with anti-psoriasin antibody (Santa Cruz Biotechnology), anti-hBD2 antibody (Santa Cruz Biotechnology), anti-KEAP1 antibody (Proteintech), anti-claudin-1 antibody (Invitrogen), anti-CD86 antibody (BD Biosciences) and anti-TLR4 (BD biosciences) at 1:200 dilutions or anti-NRF2 (Cell Signaling Technologies) at 1:100, or with anti-NOS2 antibody (Santa Cruz Biotechnology) at 1:50 dilution followed by the respective Alexa Fluor 488, Alexa Fluor 594 or Alexa Fluor 647 conjugated secondary antibody (Life Technologies) at 1:400 dilution and counter-stained using 2.5 µg mL^− 1^ 4′,6-diamidino-2-phenylindole (DAPI, Invitrogen). Confocal images were acquired on a LSM 700 microscope (Carl Zeiss) using 63× oil immersion objective. All images were processed for intensity quantification by ImageJ software (NIH).

### Western blot

T-24 cells were pretreated with 5µM of SC-236 for 24 h, followed by *E. coli* CFT073 infection for 3 h with MOI 10. After the infection time, cells were washed with 1 x PBS, 100 µl of RIPA buffer with 1% protease inhibitor cocktail (Sigma) were added and cells are scraped on ice. The cells were homogenized using a syringe and needle. The protein concentration of the samples was measured with the DC protein assay (Bio-Rad Laboratories). Equal amounts of sample were mixed with Laemmli buffer and boiled for 5 min in 95 °C. The samples (10 µg) were separated by 4–20% SDS-polyacrylamide gel electrophoresis and transferred to a polyvinylidene fluoride (PVDF) membrane (Bio-Rad Laboratories). The PVDF membrane was blocked with 3% BSA for 1 h. Caspase 1 protein was detected using a mouse monoclonal (AdipoGen Life Sciences) against human caspase 1. NLRP3 was detected using a mouse monoclonal (Abnova) against human NLRP3. GAPDH was detected with a rabbit polyclonal antibody (Santa Cruz Biotechnology Inc). All primary antibodies were incubated overnight at 4 °C. As secondary antibodies, goat anti rabbit IgG (horseradish peroxidase, HRP) (Abcam) and goat anti mouse IgG (HRP) (Abcam) were used and incubated for 1 h at room temperature. The blots were developed using Luminata Forte Western HRP Substrate (Merck Millipore).

### Caspase 1 activity assay

Bladder epithelial cells, T-24 and 5637 were grown in respective media to reach 80% confluence in a 96-well plate. Cells were pretreated with 5µM of SC-236 for 24 h and pre-incubated with 50 µM caspase 1 substrate Ac-YVAD-AMC (Enzo Life Sciences) for 1 h at 37^˚^C 5% CO_2_ prior to the *E. coli* infection. Thereafter, the cells were infected with *E. coli* at MOI 10. Samples were analyzed after 6 h of infection with a fluorescent plate reader (Cytation 3) at excitation/emission settings of 340/440 nm. Substrate with only medium was used as control to subtract the basal fluorescence later.

### Enzyme-linked immunosorbent assay

T-24, dTHP-1, NKL and dHL-60 cells were treated with 5µM of SC-236 for 36 h, followed by 2 h infection. Supernatants were collected and centrifuged at 350 g for 10 min and stored at -80 °C until needed. IL-1β (R&D Biosystems) ELISA was analyzed according to the manufacturer’s recommendations. Cells treated with DMSO were served as control.

### Statistical analysis

All statistical tests were performed in Graph pad Prism version 5. Data were obtained from Students unpaired t-test, non-parametric test using Mann Whitney u test, paired t-test and non-parametric one-way Anova, Bonferonni’s multiple comparison, Dunett’s one-way Anova test as appropriate. Differences with p values below 0.05 were considered statistically significant.

## Results

### COX-2 inhibition specifically decreases antimicrobial peptide expression

**Fig. 1:**
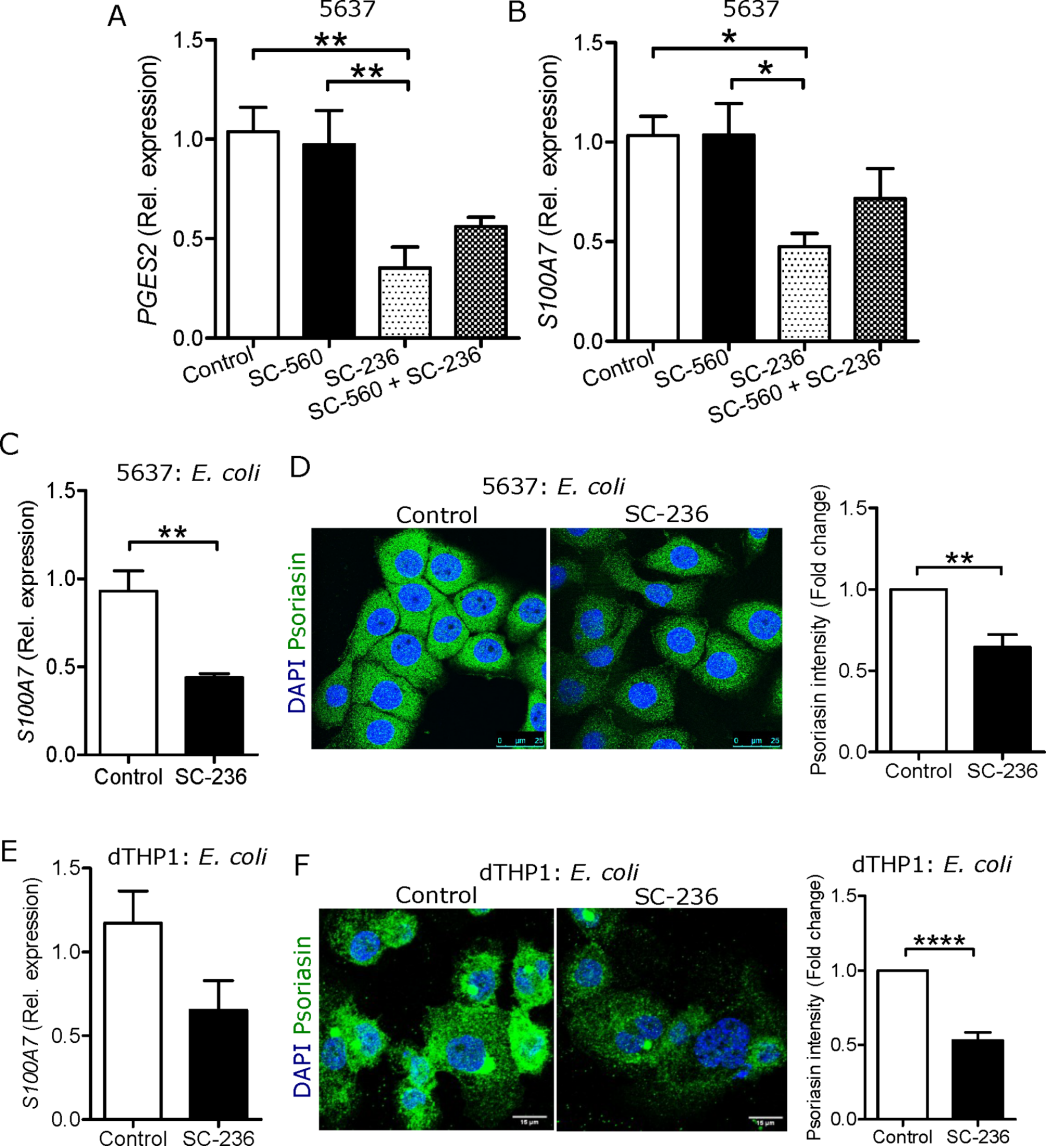
COX-2 inhibition compromised expression of the antimicrobial peptide psoriasin. Expression of **(A)***PGES2* and **(B)***S100A7* mRNA before (n = 3) and **(C)** after 15 min *E. coli* infection (n = 3) in 5637 cells. **(D)** Intracellular psoriasin stained (n = 3) with Alexa-488 (green) and DAPI (blue) for nucleus. Average fluorescence intensity of psoriasin was measured after 2 h *E. coli* infection in 5637 cells. **(E)** Expression of *S100A7* mRNA after 2 h *E. coli* infection in differentiated THP-1 (dTHP-1) cells (n = 4). **(F)** Intracellular psoriasin stained (n = 3) with Alexa-488 (green) and DAPI (blue) for nucleus, average fluorescence intensity psoriasin was measured after 30 min *E. coli* infection in dTHP-1. In vitro analysis was performed either in duplicate or triplicate. Cells were treated with 5µM SC-236 for 24 and 36 h respectively for mRNA and protein analysis followed by *E. coli* infection. Data are shown as mean ± SEM. Significance levels mentioned as *P < 0.05, **P < 0.01 and ****P < 0.0001

COX enzymes are responsible for the formation of prostaglandin E synthase 2 [23]. Therefore, we first investigated the effect of both COX-1 and 2 inhibitions in 5637 uroepithelial cells on the mRNA expression of prostaglandin E synthase 2, *PGES2.* A significant inhibition was demonstrated in the presence of COX-2 inhibitor, SC-236, whereas no difference was observed with COX-1 inhibitor, SC-560 (Fig. 1A). Likewise, treatment with the COX-2 inhibitor downregulated the AMP psoriasin, encoded by *S100A7*, in human uroepithelial cells, 5637 (Fig. 1B) and TERT-NHUC (Supplementary Fig. 2A) during non-infected state and after *E. coli* infection on both mRNA (Fig. 1C) and protein levels (Fig. 1D). Similar results were also observed for another AMP, human β defensin-2 (hBD2) in 5637 cells (Supplementary Fig. 2B-D). Therefore, we continued further with COX-2 inhibition only.

During acute infection, neutrophils contribute to the antibacterial activity by producing and releasing antimicrobial peptides [24]. Interestingly, SC-236 treatment reduced the *S100A7* mRNA levels in human primary neutrophils isolated from blood (Supplementary Fig. 2E) and DMSO differentiated promyeloblasts (dHL-60) (Supplementary Fig. 2F). Recruitment of neutrophils is dependent on the release of inflammogenic host factors from infected uroepithelial cells [24]. Therefore, we investigated the effect of conditioned media from *E. coli* infected uroepithelial T-24 cells, on the expression of psoriasin in dHL-60 cells. Stimulation of dHL-60 cells with conditioned media resulted in decreased psoriasin on the mRNA (Supplementary Fig. 2G), and increased protein levels (Supplementary Fig. 2H) compared with unstimulated cells. Macrophages, in addition to neutrophils, also play an important role in the response to *E. coli* infection [25]. In contrast to dHL-60 cells, conditioned media supplemented to THP-1 differentiated macrophages, dTHP-1, did not affect the expression of psoriasin. However, addition of SC-236 followed by *E. coli* infection in dTHP-1 macrophages showed a trend of reduced psoriasin at mRNA (Fig. 1E) but a significant reduction in protein levels (Fig. 1F).

### COX-2 inhibition prevents inflammasome mediated IL-1β secretion and activation of immune cells

**Fig. 2:**
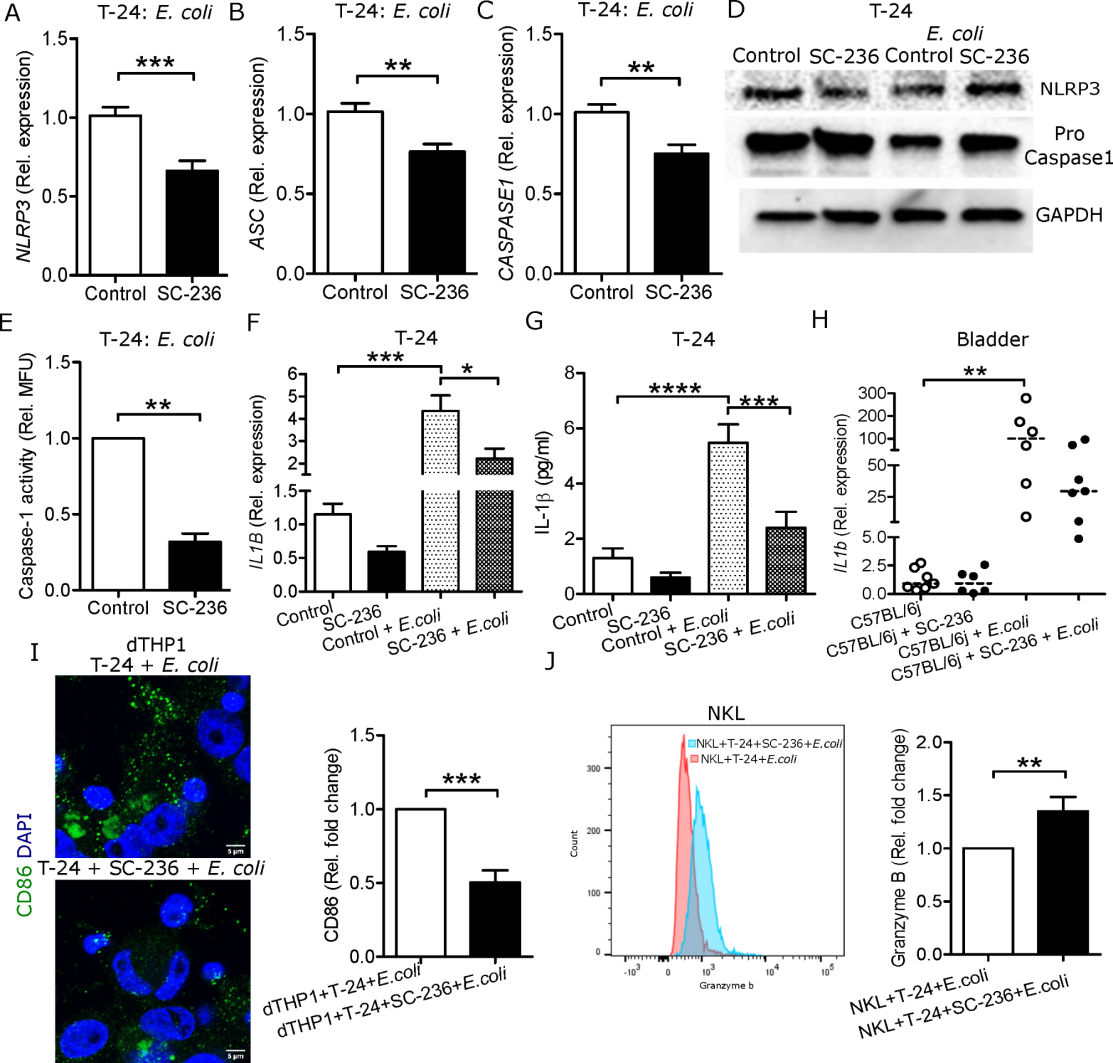
Inhibition of inflammasome pathway in COX-2 inhibited *E. coli* infected uroepithelial cells, compromised activation of immune cells. Expression of **(A)***NLRP3*, **(B)***ASC*, **(C)***CASPASE1* mRNA after 2 h *E. coli* infection (n = 4) in T-24 cells. **(D)** Representative western blot analysis of NLRP3 and Pro-caspase 1 compared to housekeeping gene, GAPDH after 3 h *E. coli* infection (n = 3). **(E)** Relative caspase 1 activity assay in T-24 cells after 6 h *E. coli* infection. Expression of the cytokine IL-1β at the **(F)** mRNA level (n = 3) and **(G)** secreted level from the supernatants using ELISA (n = 3) after 2 h *E. coli* infection in T-24 cells. **(H)** Expression of *IL1b* mRNA in the urinary bladders of SC-236 treated mice and after 24 h *E. coli* infection (n = 6/7). **(I)** Expression of CD86 in dTHP-1 cells after 2 h stimulation with conditioned media obtained from *E. coli* infected bladder epithelial cells, T-24 (n = 3) using microscopy. Relative densitometry of CD86 is shown. **(J)** Measurement of intracellular granzyme B levels in NKL cells after 6 h stimulation with conditioned media obtained from *E. coli* infected bladder epithelial cells, T-24 (n = 8). Mean fluorescence intensity (MFI) was measured and normalized to control cells. In vitro analysis was performed in either duplicate or triplicate. T-24 cells were treated with 5µM SC-236 for 24 h followed by *E. coli* infection. Conditioned media was obtained from 5µM SC-236 treated and *E. coli* infected T-24 cells after 2 h. Data are shown as mean + SEM. Results from mice are presented as median. Significance levels mentioned as *P < 0.05, **P < 0.01, ***P < 0.001 and ****P < 0.0001

Upon infection, IL-1β is produced by the inflammasome pathway [26]. *E. coli* infected SC-236 treated T-24 cells showed lower mRNA expression of the inflammasome markers *NLRP3* (Fig. 2A), *ASC* (Fig. 2B) and *CASPASE1* (Fig. 2C) compared to vehicle controls. Similarly, after 3 h of *E. coli* infection, SC-236 treated T-24 cells showed no change in NLRP3 levels or cleavage of pro-caspase 1 (Fig. 2D). Thus, indicating inhibition of the inflammasome pathway in comparison to control infected cells, whereas in uninfected cells, we did not observe any influence of SC-236 on inflammasome markers (Fig. 2D). Further to confirm, caspase 1 activity was measured in uroepithelial cells infected with *E. coli*. After 6 h of infection, caspase 1 activity was lowered in SC-236 treated cells in both the uroepithelial cells T-24 (Fig. 2E) and 5637 (Supplementary Fig. 3A).

Inhibition of caspase 1 via SC-236 resulted in lower expression of IL-1β on the mRNA (Fig. 2F) and the secreted protein after infection with *E. coli* (Fig. 2G). Similar to the in vitro results, *E. coli* infected SC-236 treated mice showed a trend of lower *Il1b* expression (Fig. 2H).

*E. coli* infection of SC-236 treated dHL-60 cells did not alter the expression of IL-1β (Supplementary Fig. 3B). However, dTHP-1 cells (Supplementary Fig. 3C) and NKL cells (Supplementary Fig. 3D) showed increased expression of IL-1β, indicating the multifaceted impact of COX-2 inhibition on various immune cells.

Immune cells are often triggered by secretory molecules released from infected uroepithelial cells thereby promoting migration to the site of infection. Therefore, activation markers were analyzed in dTHP-1 and NKL cells after treatment with conditioned media from T-24 uroepithelial cells. We observed lower expression of CD86 (Fig. 2I) in dTHP-1 cells treated with conditioned media from SC-236 and *E. coli* infected uroepithelial cells. In addition, intracellular granzyme B content was higher in SC-236 treated NKL cells in comparison with vehicle treated control cells (Fig. 2J).

### Compromised free radical formation is associated with COX-2 inhibition

**Fig. 3:**
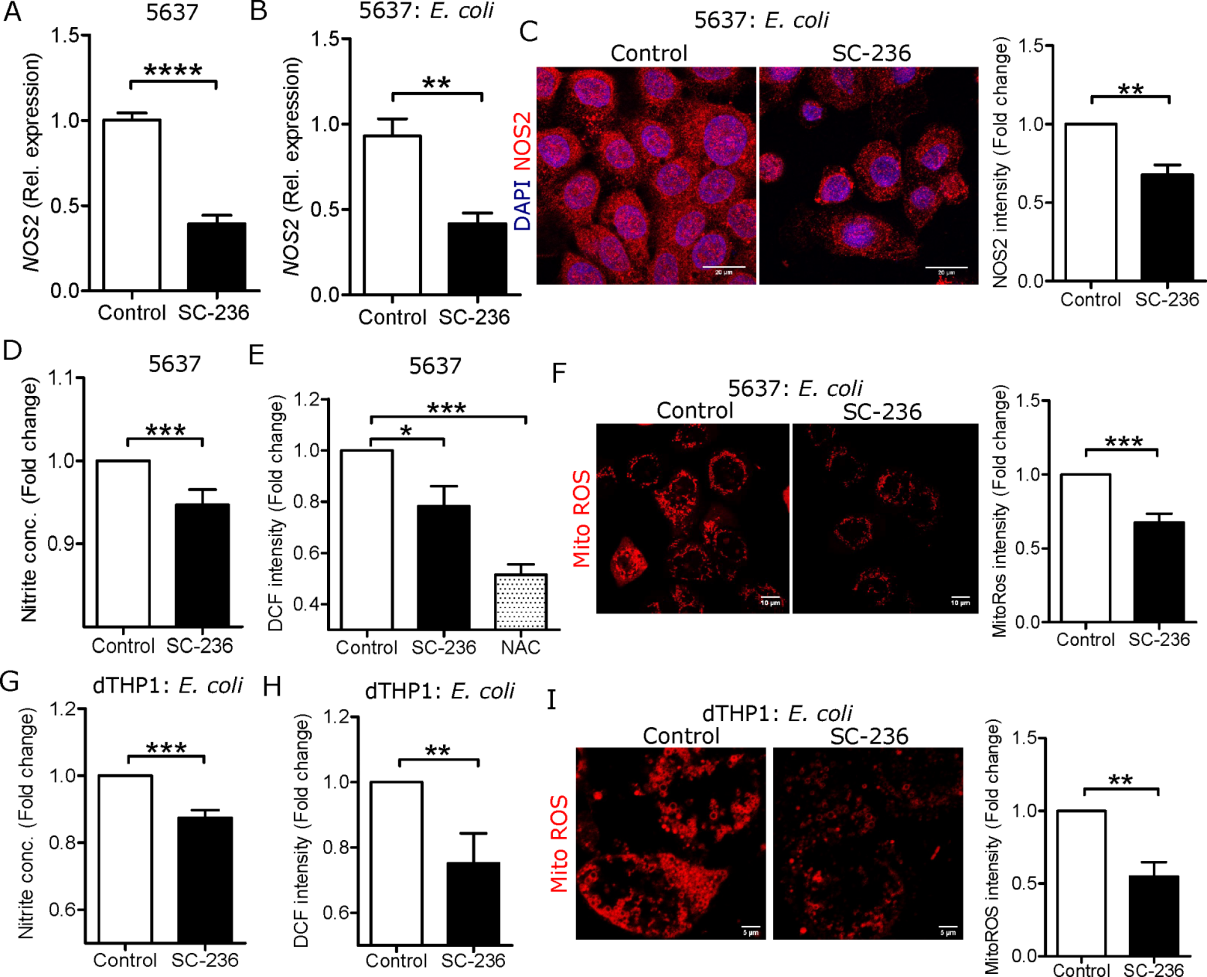
Free radical formation was reduced in COX-2 inhibited uroepithelial cells. Expression of *NOS2* mRNA **(A)** before (n = 4) and **(B)** after 15 min *E. coli* infection (n = 3) in 5637 cells. **(C)** intracellular NOS2 stained (n = 3) with Alexa-594 (red) and DAPI (blue) for nucleus, average fluorescence intensity of NOS2 was measured after 2 h *E. coli* infection in 5637 cells. **(D)** Free NO levels (n = 4) and **(E)** total intracellular ROS (n = 3) in SC-236 treated 5637 cells. **(F)** For mitochondrial ROS, mitochondria were stained after 1 h *E. coli* infection (n = 4) and fluorescence intensity was quantified. **(G)** Released free NO estimation (n = 4) and **(H)** total intracellular ROS (n = 4) in SC-236 treated dTHP-1 cells. **(I)** For mitochondrial ROS, mitochondria were stained after 1 h *E. coli* infection (n = 3) and fluorescence intensity was quantified in dTHP-1 cells were treated with 5µM SC-236 for 24 h followed by *E. coli* infection. In vitro analysis was performed in triplicate. 5µM SC-236 was treated for 24 and 36 h for mRNA and protein analysis respectively followed by *E. coli* infection. Data are shown as mean ± SEM. Significance levels mentioned as *P < 0.05, **P < 0.01, ***P < 0.001 and ****P < 0.0001

During infection, proinflammatory cytokines influence the release of free radicals [27]. Therefore, we investigated the effect of COX-2 inhibition on free radicals. Interestingly, *NOS2* mRNA was downregulated in SC-236 treated 5637 cells (Fig. 3A) and remained reduced after *E. coli* infection both on mRNA (Fig. 3B) and protein levels (Fig. 3C). In agreement with the mRNA levels, lower levels of free NO were observed in SC-236 treated 5637 cells (Fig. 3D). In addition to the reduction in NO, total intracellular ROS was also downregulated in SC-236 treated 5637 cells (Fig. 3E). Similarly, after *E. coli* infection and SC-236 treatment of 5637, a significant reduction in mitochondrial ROS was observed (Fig. 3F). To verify whether this observation was limited to uroepithelial cells only, dTHP-1 cells were analyzed showing lower NO (Fig. 3G) and total intracellular ROS (Fig. 3H) and lower mitochondrial ROS (Fig. 3I) in SC-236 treated cells compared with untreated cells.

**Fig. 4:**
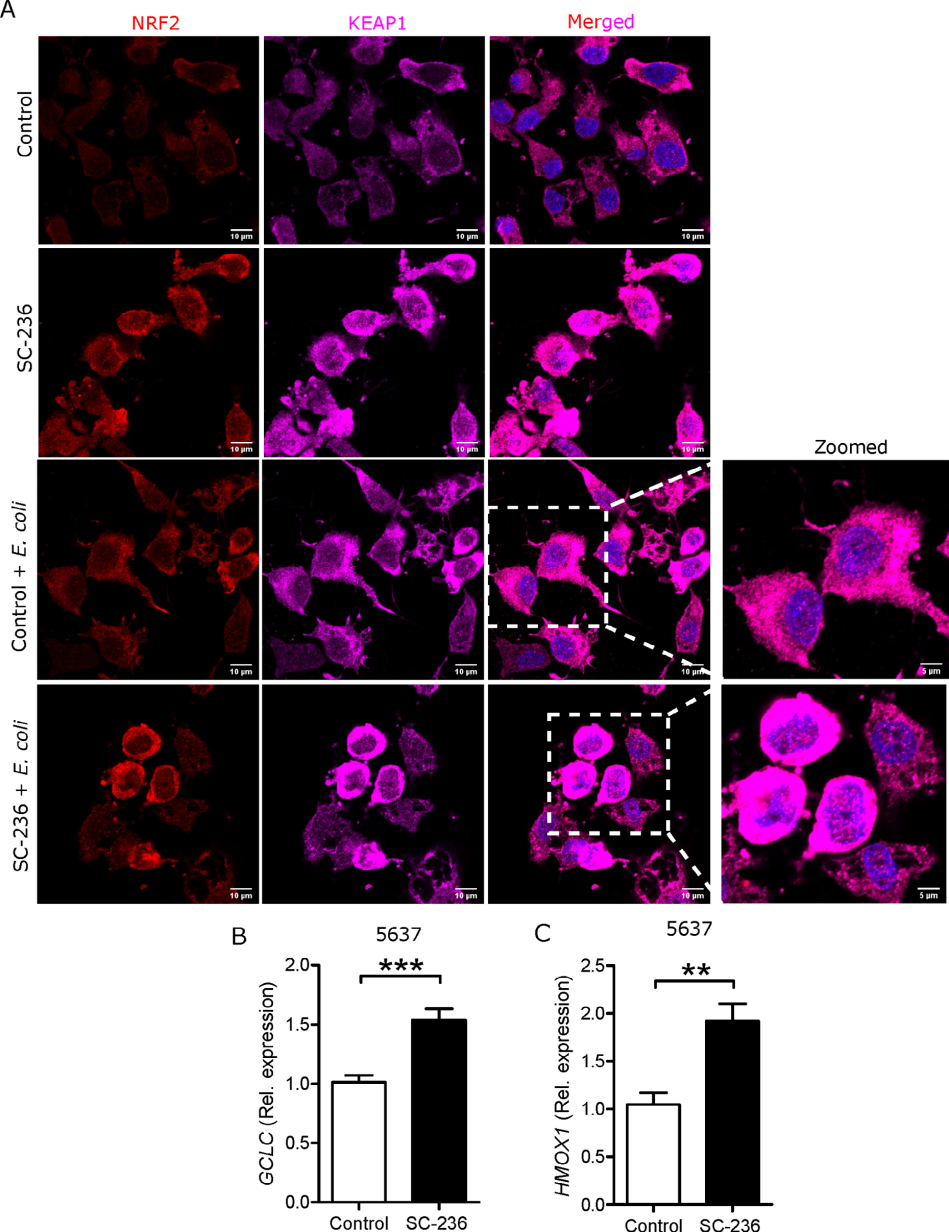
COX-2 inhibition increased antioxidant expression in uroepithelial cells. Representative expression of NRF2 (red), KEAP1 (magenta) and DAPI (blue) before and after 3 h of *E. coli* infection (n = 3) in 5637 cells. Expression of **(B)***GCLC* and **(C)***HMOX1* mRNA (n = 3) in SC-236 treated 5637 cells. In vitro analysis was performed in triplicate. 5µM SC-236 was treated for 24 and 36 h for mRNA and protein analysis respectively followed by *E. coli* infection. Data are shown as mean + SEM. Significance levels mentioned as **P < 0.01 and ***P < 0.001

Antioxidants interact with free radicals and neutralize their effects [28]. We observed a lower expression free radical in SC-236 treated uroepithelial cells and macrophages, therefore we expected a strong effect of SC-236 on regulators of cellular resistance to antioxidants, like nuclear factor erythroid 2 related factor 2 (NRF2). Interestingly, we observed increased expression of NRF2 in SC-236 treated human uroepithelial cells, 5637 (Fig. 4A). The effect was even more prominent with nuclear translocation of NRF2 in SC-236 treated *E. coli* infected cells (Fig. 4A, zoomed images). However, we also observed increased KEAP1 in these conditions.

Furthermore, to confirm the effect of NRF2 translocation, NRF2 target genes like *GCLC* (Fig. 4B) and *HMOX1* (Fig. 4C) were investigated and found to have increased mRNA expression in SC-236 treated uroepithelial cells.

### COX-2 inhibition compromises epithelial integrity and promotes *E. coli* infection

**Fig. 5:**
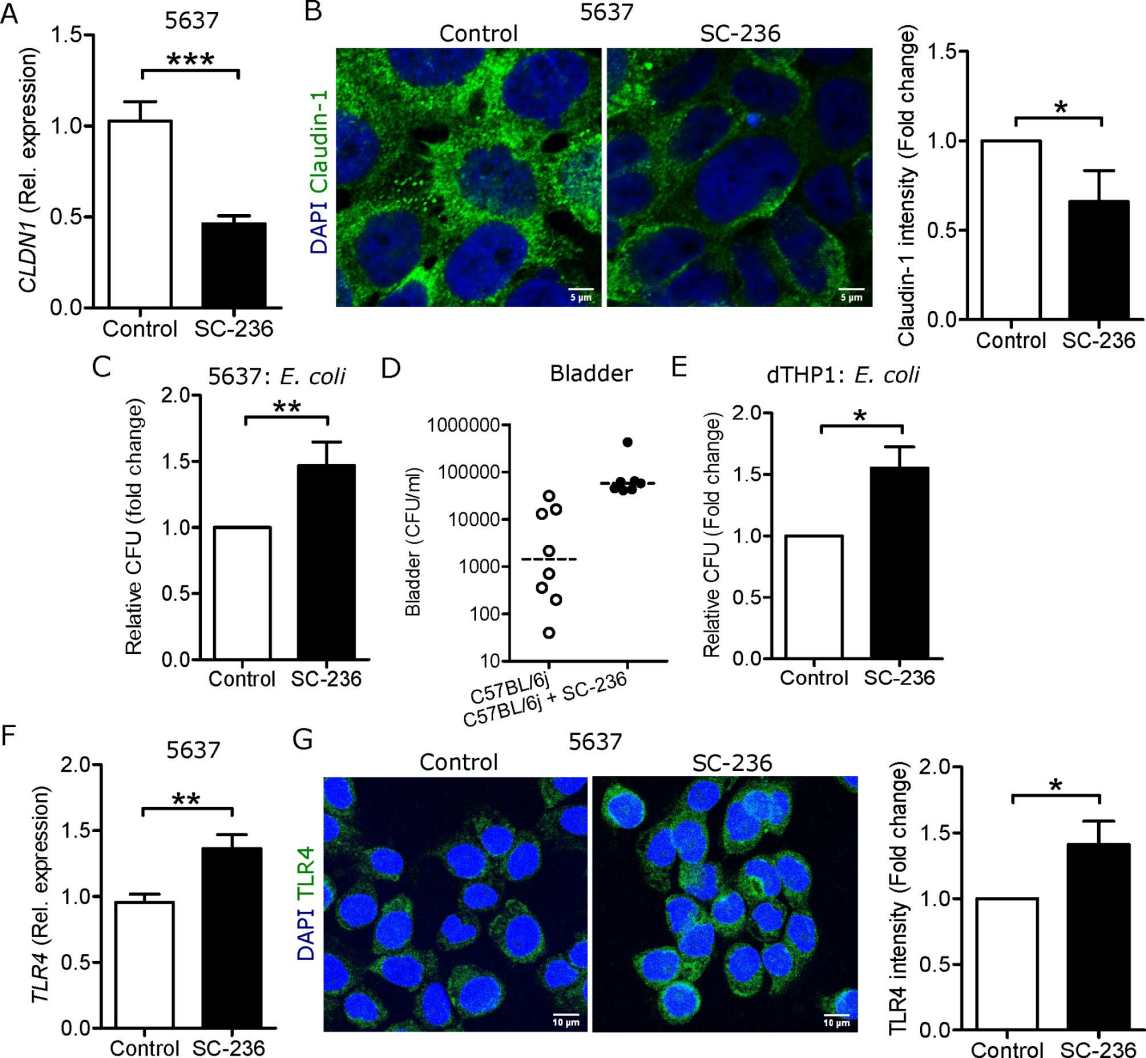
COX-2 inhibition compromised epithelial barrier and increased *E. coli* infection. Expression of claudin-1 at the **(A)** mRNA (n = 3) and **(B)** protein level (n = 3) in 5637 cells, average fluorescence intensity was measured. Intracellular *E. coli* load in **(C)** 5637 cells (n = 6), in the **(D)** urinary bladders of C57BL/6 mice (n = 8) and after treatment with SC-236 (n = 7) and **(E)** dTHP-1 cells (n = 4). **(F)** Expression of *TLR4* mRNA and **(G)** TLR4 stained (n = 3) with Alexa-488 (green) and DAPI (blue) for nucleus, average fluorescence intensity of TLR4 was measured in 5637 cells. Data are shown as mean + SEM. Results from mice are presented as median. Significance levels mentioned as *P < 0.05, **P < 0.01, and ***P < 0.001

Psoriasin is known to induce barrier proteins [29, 30], and maintain epithelial integrity. The decreased psoriasin levels seen upon treatment with COX-2 inhibition of uroepithelial cells further prompted us to investigate the effect of SC-236 on tight junction proteins. A significant reduction in the expression of claudin-1 in SC-236 treated 5637 and TERT-NHUC cells, at the mRNA (Fig. 5A and Supplementary Fig. 4A) and protein level (Fig. 5B) was observed.

Loss of epithelial integrity encouraged us to investigate the implication of *E. coli* infection in uroepithelial cells. *E. coli* survived better in SC-236 treated 5637 (Fig. 5C) and TERT-NHUC cells (Supplementary Fig. 4B). In line with our in vitro results in uroepithelial cells, bladders from mice treated with SC-236 showed limited to no clearance of *E. coli* relative to control mice, although it did not reach significance (Fig. 5D). In contrast, a significant difference was observed in dTHP-1 cells (Fig. 5E). Higher bacterial burden could be due to alteration in epithelial cell surface receptors. We therefore evaluated the expression of TLR4, responsible for the interaction with *E. coli*. Our results showed that TLR4 was upregulated both at mRNA (Fig. 5F) and protein (Fig. 5G) levels in 5637 cells after treatment with SC-236. These results could explain the increased adhesion of *E. coli* that was observed in SC-236 treated 5637 cells (Supplementary Fig. 4C).

## Discussion

We here demonstrate that the inhibition of COX-2 signaling pathway compromised the innate immune response and weakened the epithelial barrier. These findings offer an understanding of the clinical observation that NSAID treatment of patients was associated with increased pyelonephritis episodes [9–12].

Our results show that inhibition of COX-2 in uroepithelial cells mediates decreased psoriasin and hBD2 in response to *E. coli.* Inhibition of COX-2 signaling in phagocytic cells like neutrophils and macrophages resulted in compromised expression of psoriasin. These results are in line with a previous report where COX-2 expression was critical for the expression of hBD2 and hBD3 in response to *S. aureus* infection in keratinocytes [31].

Expression of the COX-2 precursor prostaglandin E2 is known to trigger the expression of antimicrobial peptides [32], it has also been shown that the antimicrobial peptide, LL-37, stimulates the activation of COX-2 signaling in keratinocytes [33]. In the context of uroepithelial cells, there are no reports suggesting the influence on antimicrobial peptides. However, we cannot completely rule out the possible interaction of psoriasin, since S100B, a member of the S100 family proteins, to which psoriasin belongs, induces COX-2 signaling in microglial cell lines [34].

The epithelial immune response is critical for effective clearance of bacteria during UTI [35]. In an autocrine pathway, IL-1β triggers the expression of COX-2 [36, 37] and cytokines like IL-8 [38, 39] which are important for the recruitment of neutrophils. Therefore, this pathway is crucial in response to invading pathogens in the urinary tract. We showed that COX-2 inhibition in uroepithelial cells compromised their IL-1β secretion, corresponding to what has been shown in ovarian granulosa cells [40]. Moreover, PGE2 is known to regulate the expression and release of cytokines particularly in innate immune cells like macrophages, neutrophils and NK cells [41]. Interestingly, when uroepithelial cells failed to release IL-1β in the presence of COX-2 inhibitor and even during *E. coli* infection, we observed increased expression of IL-1β in macrophages and NK cells which might be assumed to compensate the loss of effect in an in vivo model.

Uropathogenic *E. coli* is known to activate NLRP3 and caspase 1 mediated inflammasome in bladder epithelial cells [42]. It is also important to note that, during in vivo infection exfoliation of infected superficial bladder epithelial cells from urinary bladder is dependent on the NLRP3 inflammasome [43]. In this process, the human body clears the infected cells. Our result showing lower expression of inflammasome markers in COX-2 inhibitor treated and *E. coli* infected cells supports impediment of the inflammasome pathway. Secretion of IL-1β takes place with activation of the inflammasome [44]. The cleavage of the IL-1β precursor occurs by active caspase 1 [44]. In the present study, we observed lower caspase 1 activity in *E. coli* infected COX-2 inhibited uroepithelial cells, which resulted in compromised IL-1β levels. Altogether, our data suggests negative impact of COX-2 inhibition in uroepithelial cells. This might affect the exfoliation of infected cells in patients treated with NSAIDs, allowing the bacteria to survive longer in the host cells and be a potential threat for recurrent infection.

Activation of macrophages is important for effective phagocytic activity and bacterial clearance [45]. In agreement with this, conditioned media of COX-2 inhibited *E. coli* infected uroepithelial cells failed to fully activate macrophages, as infected uroepithelial cells release innate immune molecules to recruit innate immune cells to the site of infection. Similarly, granzyme B is an important determinant for the cytotoxicity of NKL cells, and NK cell protein levels of granzyme B can increase rapidly upon activation. Interestingly, we observed an increase in intracellular granzyme B in COX-2 inhibited NKL cells. We believe that this at least partly is a response to activating cytokines in the conditioned media. Although target cell interaction is the major trigger for granzyme B release, granzyme B can be non-specifically released after prolonged IL-2 stimulation [46]. We therefore speculate that the increased levels of granzyme B in SC-236 treated cells, could also be due to delayed release. This possibility could overall compromise the innate immunity and effective clearance of bacteria in patients treated with COX-2 inhibitors or NSAIDs.

Furthermore, COX-2 inhibited uroepithelial cells and macrophages produced less free radicals. Moreover, NO seems to interfere directly with the activity of COX-2, as production of prostaglandins by the COX-2 pathway modulates the biosynthesis of NO [47]. It has also been reported that ROS activates the expression of the COX-2 downstream protein, PGE2 [48]. Therefore, decreased levels of free radicals might further influence the COX-2 signaling pathway.

Antimicrobial peptides also largely contribute to the expression of epithelial barrier proteins. Along with psoriasin, several antimicrobial peptides, such as LL-37 and hBD2, are reported to have a beneficial effect on strengthening the epithelial barrier [29, 30, 49, 50]. Compromised psoriasin levels after COX-2 inhibition are an important observation in uroepithelial cells. It is known that COX-2 deficiency leads to epithelial barrier dysfunction [51] and application of PGE2 to ischemic-injured ileal mucosa recovered the barrier function [52]. As psoriasin is known to upregulate claudin-1 in human keratinocytes [29], we cannot rule out the indirect impact of COX-2 inhibitor, SC-236, on claudin-1 in uroepithelial cells via the psoriasin mediated pathway. Loss of epithelial integrity favors bacterial entry into the host [53]. Specifically, it has been shown that loss in claudin-1 levels resulted in increased IL-1β as well as inflammation during staphylococci infection [54].

Apart from compromising epithelial barrier function, COX-2 inhibition increased the expression of TLR4 in uroepithelial cells, which facilitated greater *E. coli* attachment. This is of high significance as PGE2 is also known to downregulate the expression TLR4 [55]. As COX-2 inhibition downregulated PGE2 in uroepithelial cells, the increase in TLR4 levels is expected and as a consequence, resulted in greater attachment of *E. coli* to uroepithelial cells.

Overall, we conclude that COX-2 inhibition compromises parameters of the innate immune response including the production of antimicrobial peptides, pro-inflammatory cytokines, epithelial barrier integrity and free radicals. Alterations in the prostaglandin receptor may influence the innate immune response status as inhibition of COX-2 creates a favorable condition for bacterial attachment and intra-cellular survival. Our data suggest that balanced COX-2 signaling is vital for the innate immune response against invading pathogens. Therefore, patients with lower UTI given NSAIDs to help treat inflammation and the pain during voiding, might be at risk of an iatrogenic-derived severe infection.

### Electronic supplementary material

Below is the link to the electronic supplementary material.

Supplementary Material 1

## Data Availability

All the raw files and other relevant information are submitted in Karolinska Institutet Electronic Lab Notebook and may be provided from corresponding author upon reasonable request.
