# Supplementary material for: Inhibition of COX-2 signaling favors E. coli during urinary tract infection
Source: J Inflamm (Lond). 2023 Sep 11;20:30. doi: 10.1186/s12950-023-00356-9 (PMC10496388; doi:10.1186/s12950-023-00356-9)
Supplement: Supplementary file 1 — Supplementary Material 1 [file 12950_2023_356_MOESM1_ESM.docx]

***Supplementary file***

**Inhibition of COX-2 signaling favors *E. coli* during urinary tract infection.**

Soumitra Mohanty^1,2^, Ciska Lindelauf^1,2^, John Kerr White^1,2^, Andrea Scheffschick^3,4^, Ewa Ehrenborg^5^_,_ Isak Demirel^6^, Hanna Brauner^3,4,7^, Annelie Brauner^1,2*^_._

^1^ Department of Microbiology, Tumor and Cell Biology, Karolinska Institutet, Stockholm, Sweden.

^2^ Division of Clinical Microbiology, Karolinska University Hospital, Stockholm, Sweden.

^3^ Department of Medicine, Solna, Stockholm, Sweden

^4^ Center for Molecular Medicine, Karolinska Institutet, Solna, Sweden

^5^ Cardiovascular Medicine Unit, Department of Medicine, Center for Molecular Medicine at BioClinicum, Karolinska University Hospital, Karolinska Institutet, Stockholm Sweden

^6^ iRiSC - Inflammatory Response and Infection Susceptibility Centre, Faculty of Medicine and Health, School of Medical Sciences, Örebro University, Örebro, Sweden.

^7^ Dermato-Venereology Clinic, Karolinska University Hospital, Stockholm, Sweden.

* Corresponding author:

Annelie Brauner, Department of Microbiology, Tumour and Cell Biology, Division of Clinical Microbiology, Karolinska Institutet and Karolinska University Hospital, 17176 Stockholm, Sweden; Phone +46 8 51770000, Fax: +46 8 308099

E-mail: Annelie.Brauner@ki.se


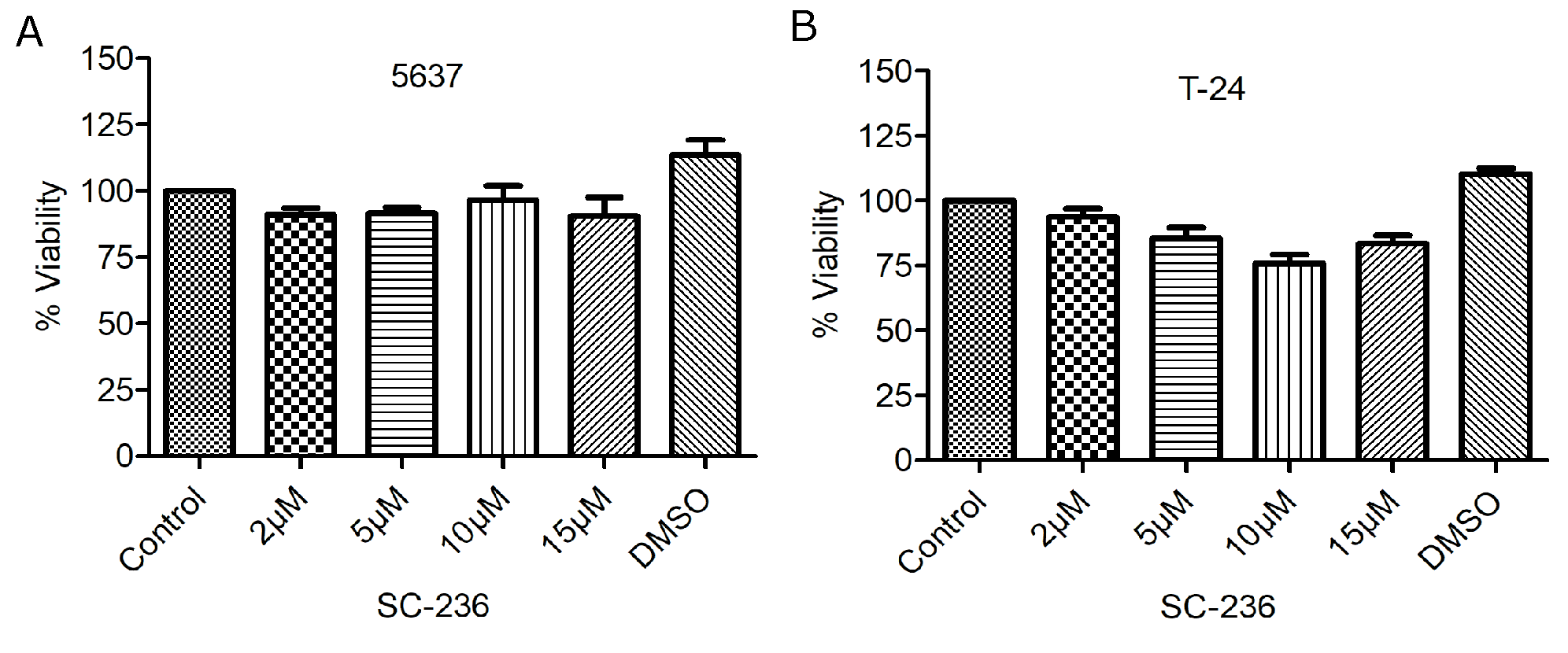


**Supplementary fig 1. Effect of COX-2 inhibitor on uroepithelial cell viability.** Metabolic activity and cell viability analyzed with XTT assay in COX2 inhibitor, SC-236 treated (A) 5637 and (B) T-24 cells at 24h (n=3). Data are shown as mean + SEM.


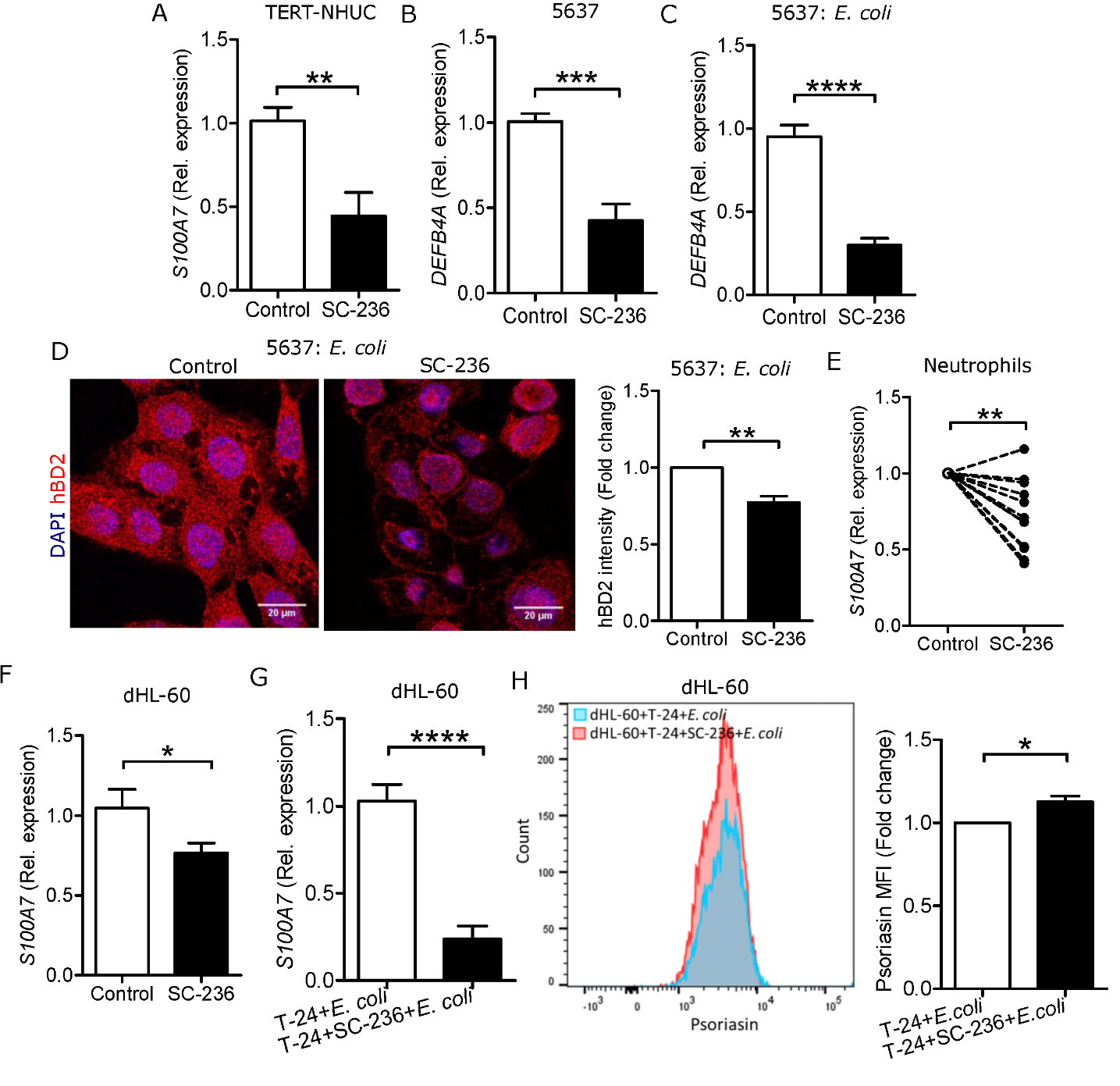


**Supplementary fig 2. COX-2 inhibition compromised expression of antimicrobial peptides.** (A) Expression of *S100A7* mRNA in TERT-NHUC cells (n=3). Expression of *DEFB4A* mRNA (B) before (n=4) and (C) after 15 mins *E. coli* infection (n=4) in 5637 cells. (D) Intracellular hBD2 stained (n=3) with Alexa-594 (red) and DAPI (blue) for nucleus, average fluorescence intensity psoriasin was measured after 2 h *E. coli* infection in 5637 cells. Expression of *S100A7* mRNA in (E) primary human neutrophils (n=12), (F) differentiated HL-60 cells (dHL-60), (n=3). (G) dHL-60 cells stimulated with *E. coli* conditioned media from T24 cells, *S100A7* mRNA levels were measured after 30 mins stimulation. (H) Flowcytometric analysis of psoriasin expression in dHL-60 cells stimulated with *E. coli* conditioned media from T24 cells (n=3), representative densitometry is presented. *In vitro* analysis was performed in either duplicate or triplicate. 5µM SC-236 was treated for 24 and 36 h respectively for mRNA and protein analysis followed by *E. coli* infection. Data are shown as mean + SEM. Significance levels mentioned as **P* < 0.05, ***P* < 0.01, ****P* < 0.001 and *****P* < 0.0001.





**Supplementary fig 3. *E. coli* infection in COX-2 inhibited immune cells differentially regulate IL-1β expression.** (A) Relative caspase1 activity assay in 5637 cells after 6 h *E. coli* infection (n=2). Estimation of IL-1β in the cell free supernatants of (B) dHL-60 (n=3), (C) dTHP1 (n=4) and (D) NKL (n=3) cells. *In vitro* analysis was performed in triplicate. Cells were treated with 5µM SC-236 for 36 h followed by 2 h *E. coli* infection. Data are shown as mean + SEM. Significance levels mentioned as ***P* < 0.01, ****P* < 0.001 and *****P* < 0.0001.





**Supplementary fig 4. COX-2 inhibition compromised claudin1 and increased *E. coli* survival.** (A) Expression of *CLDN1* mRNA in TERT-NHUC cells (n=3). (B) Intracellular *E. coli* load in TERT-NHUC (n=4) after treatment with SC-236. (C) *E. coli* rate of adhesion (n=4) in 5637 cells. *In vitro* analysis was performed in either duplicate or triplicate. 5µM SC-236 was treated for 24 h for both mRNA and *E. coli* infection. Significance levels mentioned as **P* < 0.05 and *****P* < 0.0001.

**Supplementary table 1.**

| **Gene name** | **Sequence (5’-3’)** |
| --- | --- |
| **Primers** |  |
| Human *S100A7* (Forward) | CACCAGACGTGATGACAA |
| Human *S100A7* (Reverse) | GGCTATGTCTCCCAGCAA |
| Human *DEFB4A* (Forward) | CCCTTTCTGAATCCGC |
| Human *DEFB4A* (Reverse) | GAGGGTCTTGTATCTCCT |
| Human *NLRP3* (Forward) | TGAAGAAAGATTACCGTAAGAAGTACAGA |
| Human *NLRP3* (Reverse) | GCGTTTGTTGAGGCTCACACT |
| Human *ASC* (Forward) | CGCGAGGGTCACAAACGT |
| Human *ASC* (Reverse) | TGCTCATCCGTCAGGACCTT |
| Human *CASPASE1* (Forward) | TCCCTAGAAGAAGCTCAAAGGATATG |
| Human *CASPASE1* (Reverse) | CGTGTGCGGCTTGACTTG |
| Human *IL1B* (Forward) | CACGATGCACCTGTACGATCA |
| Human *IL1B* (Reverse) | GTTGCTCCATATCCTGTCCCT |
| Mouse *Il1b* (Forward) | TGGAAAAGCGGTTTGTCT |
| Mouse *Il1b* (Reverse) | ATAAATAGGTAAGTGGTTGCC |
| Human *GCLC* (Forward) | CCCCTCCTCCAAACTCAGAC |
| Human *GCLC* (Reverse) | GCAGTACCACAAACACCACA |
| Human *HMOX1* (Forward) | CTTTTCAGAAGGGCCAGGTGA |
| Human *HMOX1* (Reverse) | GTAGACAGGGGCGAAGACTG |
| Human *CLDN1* (Forward) | TCCACTGAACAAAACCTACGC |
| Human *CLDN1* (Reverse) | TGAAAAGCAACACCAAAACG |
| Human *ACTB* (Forward) | AAGAGAGGCATCCTCACCCT |
| Human *ACTB* (Reverse) | TACATCGCTGGGGTGTTG |
| Mouse *Actb* (Forward) | CTGTCCCTGTATGCCTCTG |
| Mouse *Actb* (Reverse) | ATGTCACGCACGATTTCC |
| Human *HPRT* (Forward) | ATGGACAGGACTGAACGTCTTGC |
| Human *HPRT* (Reverse) | GACACAAACATGATTCAAATCCCTGA |
| **Probes** |  |
| *NOS2* | [Hs01075529_m1](https://www.thermofisher.com/taqman-gene-expression/product/Hs01075529_m1?CID=&ICID=&subtype=) |
| *TLR4* | [Hs00152939_m1](https://www.thermofisher.com/taqman-gene-expression/product/Hs00152939_m1?CID=&ICID=&subtype=) |
| *GAPDH* | 4326317E |

**Supplementary full blot image for Figure 2 D.**

**
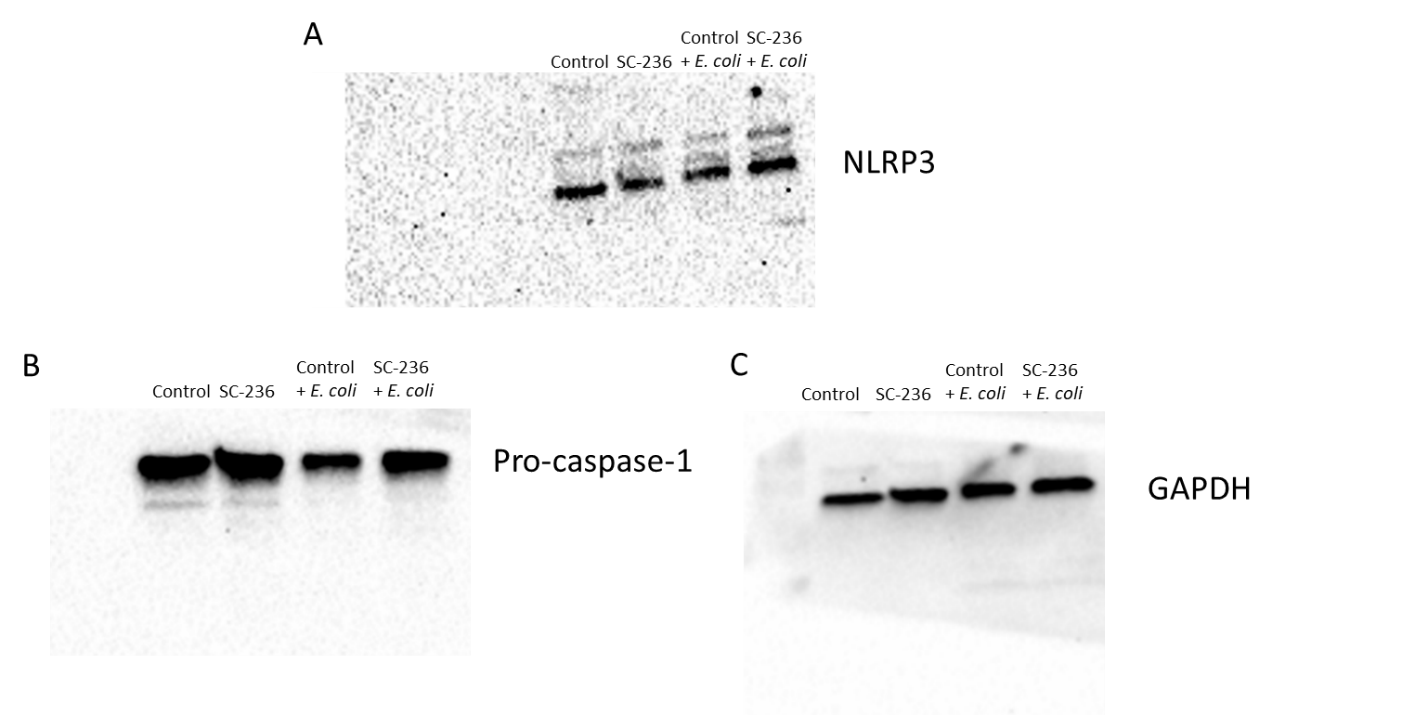
**

Supplementary full western blot images for (A) NLRP3, (B) Pro-caspase-1 and (C) GAPDH.
